# Survival and Mid-Term Outcomes of On Pump *vs.* Off Pump Coronary Artery Bypass Grafting: A Propensity Score-Matched Analysis in A First Peruvian Registry.

**DOI:** 10.21470/1678-9741-2023-0242

**Published:** 2024-11-18

**Authors:** W Samir Cubas, Wildor Dongo-Minaya, Franco Albán-Sánchez, Jose Torres-Neyra, Anna Paredes-Temoche, Katherine Inga-Moya, Hector Bedoya-Copello, Wilfredo Luna-Victoria, Enrique Velarde-Revilla

**Affiliations:** 1 Department of Thoracic and Cardiovascular Surgery, Heart Surgery Service, Edgardo Rebagliati Martins National Hospital, Lima, Peru; 2 Alberto Hurtado Medical School, Cayetano Heredia Peruvian University, Lima, Peru

**Keywords:** Coronary Artery Bypass, Extracorporeal Membrane Oxygenation, Coronary Disease, Survival, Propensity Score

## Abstract

**Introduction:**

The efficacy and outcomes of on-pump and off-pump coronary artery bypass grafting (CABG) remain uncertain, especially in Latin America. Our study aims to explore survival and shortand mid-term outcomes in the first reported Peruvian registry of patients treated with both techniques.

**Methods:**

This is an observational, analytical, and longitudinal study using a propensity score-matched (PSM) analysis in a single-center retrospective registry of 2280 patients during 2000-2019; 846 patients were analyzed after PSM (on-pump = 423 vs. off-pump = 423). Baseline variables, comorbidities, and major outcomes were studied in the short term (≤ 30 days) and in midterm (30 days-36 months) with major adverse cardiac and cerebrovascular events. The matched groups were compared by descriptive, multivariate, and Kaplan-Meier survival analyses.

**Results:**

Before PSM, previous myocardial infarction < 7 days (27.03%) and ejection fraction ≥ 50% (45.72%) were higher in off-pump CABG (P<0.05). After PSM, pre-surgery percutaneous coronary intervention (27.18% vs. 26.71%, P=0.049) and Society of Thoracic Surgeons risk score (1.98% vs. 1.90%, P=0.047) were higher in off-pump CABG. In the short term, there was higher mortality (2.12% vs. 0.47%, P=0.048), blood transfusion > 500 ml (57.91% vs. 7.56%, P=0.049), reintervention (7.32% vs. 2.12%, P=0.045), hospital stay (nine vs. four days, P=0.048), arrhythmia (9.92% vs. 4.96%, P=0.049), and renal failure (20.09% vs. 5.91%, P=0.009) in on-pump CABG. Long-term mortality (4.25% vs. 1.65%, P=0.044), myocardial infarction (17.02% vs. 7.32%, P=0.046), and repeat revascularization (17.49% vs. 8.26%, P=0.045) predominated in on-pump CABG. There was a higher 36-month adjusted survival for off-pump over on-pump CABG (97.88% vs. 93.63%, P=0.046).

**Conclusion:**

This first reported Peruvian registry of patients treated with CABG has demonstrated that off-pump CABG is associated with lower shortand mid-term morbidity and mortality rates and better-adjusted survival rates compared to on-pump CABG; however, further multicenter studies in Latin America are needed to elucidate its benefits over classic on-pump CABG.

## INTRODUCTION

**Table:** 

Abbreviations, Acronyms & Symbols				
BMI	= Body mass index		ICU	= Intensive care unit
CABG	= Coronary artery bypass grafting		MACCE	= Major adverse cardiac and cerebrovascular events
CAD	= Coronary artery disease		MI	= Myocardial infarction
CI	= Confidence interval		OR	= Odds ratio
CKD	= Chronic kidney disease		PCI	= Percutaneous coronary intervention
COPD	= Chronic obstructive pulmonary disease		PSM	= Propensity score-matched
ECC	= Extracorporeal circulation		SD	= Standard deviation
eGFR	= Estimated glomerular filtration rate		STS	= Society of Thoracic Surgeons
HR	= Hazard ratio			

Coronary artery bypass grafting (CABG) remains the gold standard therapy for revascularization of multivessel coronary artery disease (CAD) in the presence of complex coronary anatomy^[1-3]^. This procedure is conventionally performed with extracorporeal circulation (ECC) support and is often referred to as on-pump CABG. The use of ECC has been associated with a series of multi-organ physiological alterations and contributes to associated morbidity and mortality so coronary revascularization without the use of ECC has been developed to reduce associated perioperative complications and improve shortand mid-term results^[4-6]^. However, studies have not been able to definitively elucidate any consistent advantage of off-pump over on-pump CABG, and among the factors to be considered for the success of this technique are the surgeon’s expertise on the beating heart and the use of cardiac stabilization devices during the procedure. Recently, several randomized controlled trials with a large number of patients have reported encouraging comparable results between both techniques despite the negative publicity and latent concern about worse survival and incomplete revascularization associated with off-pump CABG^[3-5]^.

The persistent skepticism regarding this technique, especially in Latin America, has prompted us to review and compare the survival and mid-term results between on-pump *vs.* off-pump CABG in the first Peruvian registry of patients who underwent these techniques.

## METHODS

### Design and Sample Size

This is an analytical, longitudinal, and retrospective study. All patients with CAD and who underwent CABG by our cardiac surgery service during the period 2000-2019 were included. Our service (Edgardo Rebagliati Martins National Hospital) is one of the leading centers nationwide in the surgical treatment of coronary pathology and has > 12% of the national affiliated population. All patients with on-pump and off-pump CABG were enrolled in the study and had complete preoperative, intraoperative, and postoperative records up to three years after hospital discharge analyzed.

### Off-Pump CABG

This technique requires cardiac manipulation while maintaining hemodynamic stability and is performed with the use of suction and stabilization devices. It is performed by a complete sternotomy, and manipulation of the heart is facilitated using suction devices. A soft cup device is usually used at the apex of the left ventricle to allow movement of the heart for manipulation. The target vessel is further immobilized using another plate device with multiple suction cups lateral to the target vessel. Both suction devices are attached to the sternal retractor and can be adjusted as needed. The target vessel is immobilized to facilitate anastomosis, and the bypass grafts must be sutured while the heart is beating and the lungs are actively ventilating. The patient is usually placed in the Trendelenburg position to facilitate venous return to the heart. In addition, devices are used to visualize the open target vessel during anastomosis and these tools include proximal occlusion strips or intracoronary shunts, as well as carbon dioxide gas and saline perfusate devices. Coronary proximal occlusion requires a period of ischemia while the anastomosis is being performed, whereas the use of an intracoronary shunt does not. Proximal graft anastomoses can be performed on internal mammary artery grafts or on the ascending aorta with the use of a partial aortic clamp or a proximal anastomosis device that does not use clamping.

### Data Collection and Study Variables

The main sources of information were the electronic medical record, operative reports, and the outpatient evaluation form. We initially identified all patients seen by our service with the designation “coronary artery bypass graft carrier”, and who were subsequently enrolled for the study only during the study period. Data were collected retrospectively and longitudinally, selected and organized according to the chronology of hospital care for three years after surgical treatment with CABG.

The main variables were divided into two main groups considering the type of CABG technique (on-pump *vs.* off-pump) and the propensity score-matched (PSM) analysis. The PSM analysis considered the baseline characteristics such as age, gender, body mass index (BMI), hemoglobin, and glomerular filtration rate; comorbidities that mainly included diabetes, hypertension, dyslipidemia, chronic obstructive pulmonary disease, and stroke, history of myocardial infarction (MI), ejection fraction, number of coronary vessels affected, and postoperative mortality calculated with the Society of Thoracic Surgeons (STS) score were also considered. Outcomes were perioperative or short-term (≤ 30 days) based on mortality, blood transfusion, surgical reintervention, postoperative arrhythmias, and acute renal failure, and midterm (30 days-3 years) based on major adverse cardiac and cerebrovascular events (MACCE) such as mortality, MI, stroke, and need for repeat revascularization.

The choice of the type of CABG was decided by the surgical team in charge of the case, considering experience with one or another revascularization technique, baseline characteristics of the patient, data on the risk of perioperative mortality, and also taking into account the patient’s decision on his/her condition and the therapeutic option.

### Statistical Analysis

Categorical variables were presented as frequencies and percentages, and continuous variables as means ± interquartile ranges - in some cases, with standard deviation. Scores were evaluated using PSM models to compare on-pump *vs.* off-pump CABG variables. After weighting, the imbalance was minimal with all standardized differences ≤ 10% and these data were used to create inverse probability weights. The data were then reweighted to ensure that the distribution of confounders and confounders was equal between the two comparison groups. All relevant confounders were adjusted for, preserving the entire sample size and ensuring that the results of the study were more generalizable.

The matched groups were compared by descriptive (unpaired Student’s *t*-test if the distribution was normal and Mann-Whitney U test if it was not), multivariate, and Kaplan-Meier survival analyses. In all cases, the data collection, tabulation, and analysis were performed using the statistical software Stata, version 16 (StataCorp LLC, College Station, Texas) for Windows version 10. The statistical findings were considered significant with a value of *P*<0.05.

### Ethical Aspects

The study protocol was approved by the Cardiac Surgery Department and the Hospital Ethics Committee (HNERM_04/CE_RAR23). The guidelines proposed by the Declaration of Helsinki were followed, data confidentiality was respected and informed consent was not required due to the type of retrospective study.

## RESULTS

**Fig. 1:**
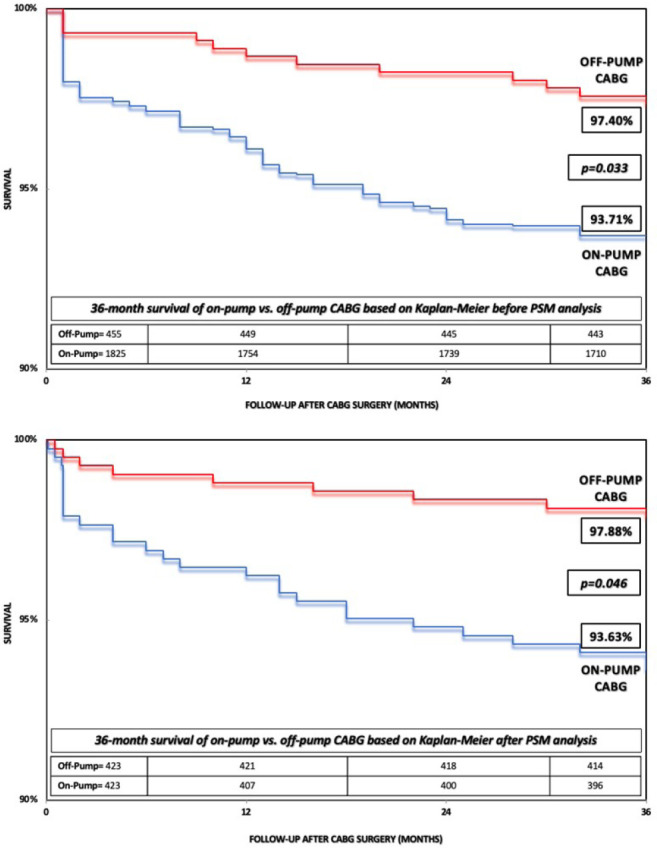
A) Kaplan-Meier 36-month survival curve of patients undergoing on-pump vs. off-pump coronary artery bypass grafting (CABG) before propensity score-matched (PSM) analysis. B) Kaplan-Meier 36-month survival curve of patients undergoing on-pump vs. off-pump CABG after PSM analysis.

**Fig. 2:**
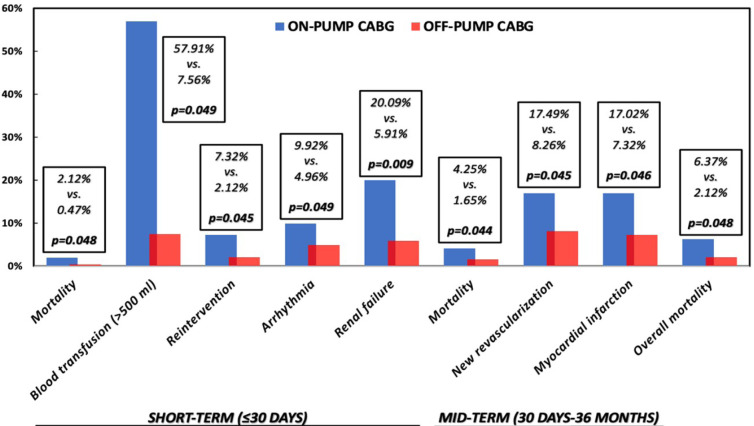
Shortand mid-term outcomes studied in patients undergoing on-pump vs. off-pump coronary artery bypass grafting (CABG).

Data from 2280 patients undergoing on-pump *vs.* off-pump CABG were evaluated. After applying the PSM models (on-pump = 423 *vs.* off-pump = 423), the mean age was 65.32 years, with > 50% being male (56.74%) and > 30% of patients with a BMI > 30 kg/m^2^. The main comorbidities, by a notable difference, were diabetes mellitus 2 (39%), arterial hypertension (32%), and dyslipidemia (27%). Before PSM, previous MI < 7 days (on-pump 13.31% *vs.* off-pump 27.03%) and ejection fraction ≥ 50% (on-pump 38.47% *vs.* off-pump 45.72%) were higher in the off-pump group presenting statistical significance (*P*<0.05). However, after PSM, the percentage of patients presenting ejection fraction ≥ 50% increased (on-pump 49.89% *vs.* off-pump 52.49%), and in the case of previous MI < 7 days, the prevalence was reversed with higher values in the on-pump group (on-pump 21.04% *vs.* off-pump 15.83%). After PSM, preoperative percutaneous coronary intervention (PCI) (27.18 % *vs.* 26.71 %, *P*=0.049) and STS risk score (1.98 % *vs.* 1.90 %, *P*=0.047) were higher in the off-pump mode; however, this statistical significance and prevalence of higher values in the off-pump group were present before performing PSM. In the short-term, there was higher mortality (2.12% *vs.* 0.47%, *P*=0.048), blood transfusion > 500 ml (57.91% *vs.* 7.56%, *P*=0.049), reoperation (7.32% *vs.* 2.12%, *P*=0.045), hospital stay (nine *vs.* four days, *P*=0.048), arrhythmia (9.92% *vs.* 4.96%, *P*=0.049), and renal failure (20.09% *vs.* 5.91%, *P*=0.009) in the on-pump group (Figures 1 and 2). Longer hospital stays and complications would be mainly due to the greater manipulation of the aorta involved in on-pump surgery. Regarding variables after 30 days, long-term mortality (4.25% *vs.* 1.65%, *P*=0.044), MI (17.02% *vs.* 7.32%, *P*=0.046), and need for new revascularization (17. 49% *vs.* 8.26%, *P*=0.045), including PCI (13.71% *vs.* 6.14%, *P*=0.049) and CABG (3.78% *vs.* 2.12%, *P*=0.041), presented a greater predominance in the on-pump group. Furthermore, of the total number of patients who presented a need for new revascularization, the majority underwent PCI (77%) (Table 1).

**Table 1:** Baseline, clinical-surgical, and outcome characteristics of patients undergoing on-pump *vs.* off-pump CABG.

Variables	Baseline characteristics of patients before PSM analysis (n=2280)			Baseline characteristics of patients after PSM analysis (n=846)		
	On-pump (n=1825) N (%)	Off-pump (n=455) N (%)	*P*-value	On-pump (n=423) N (%)	Off-pump (n=423) N (%)	*P*-value
Age ± SD (years)	64.76 ± 8.56	68.98 ± 6.32	0.098	65.53 ± 7.34	65.12 ± 9.09	0.108
Male sex	1123 (61.53)	231 (50.76)	0.087	241 (56.97)	239 (56.50)	0.091
BMI ≥ 30 kg/m^2^	657 (36)	143 (31.42)	0.093	123 (29.07)	138 (32.62)	0.110
Hemoglobin ± SD (mg/dl)	12.01 ± 1.83	12.85 ± 1.21	0.101	11.98 ± 1.92	11.91 ± 1.56	0.134
eGFR ± SD (mg/dl)	95.67 ± 23.09	91.14 ± 17.24	0.075	86.89 ± 17.14	89.01 ± 19.45	0.089
Comorbidities						
Diabetes mellitus 2	654 (35.83)	197 (43.29)	0.099	165 (39)	169 (39.95)	0.123
Hypertension	467 (25.58)	145 (31.86)	0.081	132 (31.20)	139 (32.86)	0.090
Dyslipidemia	356 (19.50)	122 (26.81)	0.068	119 (28.13)	113 (26.71)	0.072
COPD	123 (6.73)	76 (16.70)	0.051	56 (13.23)	49 (11.58)	0.059
CKD	119 (6.52)	69 (15.16)	0.058	49 (11.58)	46 (10.87)	0.068
Smoking	125 (6.84)	58 (12.74)	0.063	51 (12.05)	48 (11.34)	0.093
Stroke	98 (5.36)	33 (7.25)	0.082	29 (6.85)	31 (7.32)	0.099
Drug addiction	76 (4.16)	26 (5.71)	0.079	22 (5.20)	27 (6.38)	0.083
Prior myocardial infarction						
< 7 days	243 (13.31)	123 (27.03)	0.045	89 (21.04)	67 (15.83)	0.051
≥ 7 days	237 (12.98)	87 (19.12)	0.065	69 (16.31)	103 (24.34)	0.080
Ejection fraction						
< 50	1123 (61.53)	247 (54.28)	0.078	212 (50.11)	201 (47.51)	0.097
≥ 50	702 (38.47)	208 (45.72)	0.049	211 (49.89)	222 (52.49)	0.059
Diseased vessels						
Left main stenosis	454 (28.87)	141 (30.98)	0.061	139 (32.86)	130 (30.73)	0.095
1-3	885 (48.49)	187 (41.09)	0.082	165 (39)	174 (41.13)	0.102
≥ 3	486 (22.64)	127 (27.93)	0.057	119 (28.14)	119 (28.14)	0.069
Pre-surgery PCI	345 (18.90)	121 (26.59)	0.036	113 (26.71)	115 (27.18)	0.049
STS risk score (%)	1.95	2.27	0.039	1.90	1.98	0.047
Outcomes						
Short-term (≤ 30 days)						
Mortality	37 (2.02)	3 (0.65)	0.029	9 (2.12)	2 (0.47)	0.048
Distal anastomoses						
1-2	789 (43.23)	198 (43.51)	0.084	138 (32.62)	147 (34.75)	0.091
≥ 3	1036 (56.77)	257 (56.49)	0.095	285 (67.37)	276 (65.24)	0.110
Bypass time/cross-clamping time (min)	99/61	0/0	0.001	92/60	0/0	0.004
Blood transfusion (> 500 ml)	1155 (63.28)	62 (13.62)	0.046	245 (57.91)	32 (7.56)	0.049
Reintervention	145 (7.94)	15 (3.29)	0.049	31 (7.32)	9 (2.12)	0.045
Hospital stay ± SD (days)	11 ± 4.56	6 ± 3.12	0.031	9 ± 3.91	4 ± 0.19	0.048
Arrhythmia	214 (11.72)	25 (5.49)	0.045	42 (9.92)	21 (4.96)	0.049
Renal failure	452 (24.76)	39 (8.57)	0.004	85 (20.09)	25 (5.91)	0.009
Stroke	98 (5.36)	10 (2.19)	0.056	21 (4.96)	9 (2.12)	0.068
Mid-term (30 days - 36 months)						
Mortality	78 (4.27)	9 (1.95)	0.038	18 (4.25)	7 (1.65)	0.044
New revascularization						
PCI	267 (14.63)	32 (7.03)	0.042	58 (13.71)	26 (6.14)	0.049
CABG	81 (4.43)	11 (2.41)	0.030	16 (3.78)	9 (2.12)	0.041
Myocardial infarction	311 (17.04)	34 (7.47)	0.035	72 (17.02)	31 (7.32)	0.046
Stroke	122 (6.68)	15 (3.29)	0.059	26 (6.14)	12 (2.83)	0.069

BMI=body mass index; CABG=coronary artery bypass grafting; CKD=chronic kidney disease; COPD=chronic obstructive pulmonary disease; eGFR=estimated glomerular filtration rate; PCI=percutaneous coronary intervention; PSM=propensity score-matched; SD=standard deviation; STS=Society of Thoracic Surgeons

**Fig. 3:**
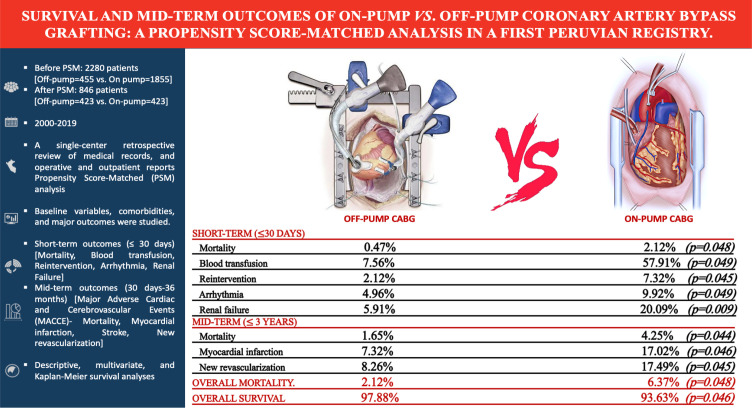
Highlights of survival and mid-term outcomes of on-pump vs. off-pump coronary artery bypass grafting (CABG): a propensity score-matched (PSM) analysis in a first Peruvian registry.

In the Kaplan-Meier analysis, a higher adjusted survival at 36 months was identified for off-pump compared to on-pump CABG with statistical significance (97.88% *vs.* 93.63%, *P*=0.046), indicating that the greater reduction in survival for the on-pump group occurs in the first 12 months, obtaining a greater survival at 36 months for the off-pump group (Figures 1-3).

## DISCUSSION

The average age of patients who underwent CABG surgery was between 65 and 69 years, and this is similar to that reported by Quin et al.^[1]^ who showed an average age of 63 years; likewise, they described that the male gender is the most frequently affected by CAD, like us. Figueroa et al.^[2]^ reported an average age of 66.7 years in patients undergoing revascularization, but unlike us, they found that the female sex is the most frequently associated with these surgical procedures (*P*=0.003). Concerning comorbidities, Wang et al.^[3]^ reported that hypertension (65%) and diabetes mellitus (76.9%) were the main conditions most associated with this CABG cohort; likewise, Orellana et al. evidenced that diabetes mellitus is a strong predictor for presenting at least one revascularization-related MACCE event (hazard ratio [HR] 3.4, 95% confidence interval [CI] 2.5-4.9, *P*=0.045)^[4]^.

We identified that about 25% of patients undergoing surgical revascularization in the pre-and post-PSM sample had at least one previous percutaneous revascularization procedure, being similar to that reported by Tena et al.^[5]^ who showed that > 80% of successful off-pump CABG cases had at least one previous transcatheter procedure with coronary stenting (*P*=0.002).

Patients who underwent off-pump CABG had a higher STS risk score than those in the comparative group and this was one of the reasons for the choice of coronary revascularization therapy, which sought to avoid aortic clamping and the complications associated with the use of ECC in patients with serious comorbidities. Sun et al.^[6]^ identified that off-pump CABG was associated with lower postoperative stroke rates (8% *vs.* 23%, *P*=0.003), due to no aortic manipulation and lower levels of inflammatory reactants associated with ECC compared to on-pump CABG (21% *vs.* 54%, *P*=0.001). Likewise, Rocha et al.^[7]^ reported lower rates of chronic kidney disease and dialysis support in the off-pump group (odds ratio [OR] 0.5, 95% CI 0.34-0.69, *P*=0.003) and it was due to the low elevation of creatinine and uremic metabolites associated with the revascularization procedure.

Shortand mid-term mortality rates were lower in the off-pump group with a statistically significant difference before and after PSM analysis. In contrast to these findings, Jacquelyn A Quin et al.^[1]^ evaluated the results of a 10-year randomized controlled trial comparing on-pump *vs.* off-pump surgery (ROOBY trial), and their analysis of 1104 patients found no statistically significant advantage (*P*=0.07). Likewise, Rösler et al. found similar 30-day mortality rates in a Brazilian population, and the difference was not significant (1.5% *vs.* 2.4%, *P*=0.401)^[8]^.

In a Korean cohort of revascularized coronary patients, no differences were found in 30-day postoperative mortality between the two surgical groups; however, at mid-term and long-term follow-ups for 5.3 years, it was determined that the off-pump CABG group was associated with a higher rate of late mortality (23% *vs.* 12%, *P*=0.02), need for new revascularization (*P*=0.001), and MI (*P*=0.22)^[9]^. However, Cheng Wang et al.^[3]^ studied a sample of 1200 patients and determined fewer postoperative complications such as renal failure (5% *vs.* 12%, *P*=0.003), respiratory failure (3.5% *vs.* 10.1%, *P*=0.001), reinterventions due to bleeding (2% *vs.* 8.3%, *P*=0.01), cerebrovascular events (5.2% *vs.* 13.2%, *P*=0.01), as well as shorter stay in the intensive care unit (ICU) (*P*=0.009) and faster recovery in patients who underwent off-pump CABG. Furthermore, paradoxically, it identified a higher rate of incomplete revascularization in the on-pump CABG group (12.4% *vs.* 8.2%, *P*=0.03), and no significant differences in early mortality were found between both groups after PSM analysis. Park et al. described that at 30 days postoperatively, there was no significant difference in adjusted mortality between both groups (HR 1.00; 95% CI 0.87-1.16, *P*=0.002) in long-term follow-up; however, off-pump surgery was associated with an increased risk of mortality (HR 1.09; 95% CI 1.03-1.15, *P*=0.001), MI (HR 1.3; 95% CI 1.16-1.45, *P*=0.02), and new revascularization (HR 1.50; 95% CI 1.37-1.63 *P*=0.003)^[10]^. Squiers et al.^[11]^ described that off-pump surgery was associated with a significant, but clinically modest, increased risk of mortality compared to on-pump surgery (5.6% *vs.* 4.8%, *P*=0.002). However, the risk was substantially reduced when off-pump surgery was performed by surgeons with a higher volume of previously performed cardiac surgeries, in other words with greater experience (> 100 cases per year).

On the other hand, Jiang et al.^[12]^ described in a review of 18 studies that patients undergoing on-pump CABG had a higher risk of stroke (23%), renal failure (34%), operative bleeding (> 500 ml), and arrhythmias (11%), all mainly due to the effect of ECC; in this regard, one of the greatest statistical differences we obtained in the short term was the risk of renal failure, which was higher in the on-pump group, coinciding with a recently published study. This showed that patients undergoing off-pump CABG reintervention had a lower rate of renal failure and need for dialysis (0% *vs.* 4.6%, *P*=0.01)^[13]^.

A study carried out in Brazil did not show significant differences between off-pump *vs.* on-pump CABG about short-term mortality, stroke, or new revascularization^[9]^; however, an Asian cohort showed a higher risk of MI and repeat revascularization in 45% of the cases of patients with off-pump CABG (*P*=0.04)^[10]^. Ali Sheikhy et al.^[14]^ studied 8163 patients and showed similar 30-day mortality rates between both groups; however, off-pump CABG had a shorter hospital stay (*P*<0.001), shorter intubation time (*P*=0.003), and shorter ICU stay (*P*<0.001). Unfortunately, off-pump CABG was associated with a higher risk of 30-day mortality in the adjusted analysis (OR 1.7; 95% CI 1.09-2.65; *P*=0.019) but after PSM, this difference was not statistically significant (*P*=0.09).

In contrast, Chan et al.^[15]^ published the results of their analysis involving 6436 octogenarian patients, in which they also applied PSM analysis and found similar results to ours on mortality (4% *vs.* 5.6%, *P*=0.001) and lower in-hospital complications for off-pump CABG such as stroke (1.4% *vs.* 4.6%, *P*=0.01); however, they showed an unusually higher need for dialysis in patients undergoing off-pump surgery (10.4% *vs.* 5.9%, *P*=0.04). Several published meta-analyses such as the one by Machado et al.^[16]^, which included patients older than 65 years, showed no statistically significant mid-term differences in mortality, MI, stroke, and renal complications; however, it highlighted that those patients undergoing off-pump CABG had higher risk of new revascularization (13.4% *vs.* 5%, *P*=0.022), this finding reinforces the idea that off-pump surgery could be associated with incomplete revascularizations as found in our work.

In another context, one of the variables that were not analyzed in the present study was graft patency compared between the two groups, but which was studied by Zhou et al.^[17]^ (off-pump = 5743 *vs.* on-pump = 5898), reporting a lower pathology if off-pump is performed in grafts in general with a higher risk of obstruction such as those of the greater saphenous vein and in the territories of the left coronary artery (HR 1.31; 95% CI 1.01-1.62, *P*=0.006); however, no differences were found comparing the groups in arterial grafts and the territory of the right coronary artery.

About follow-up studies in this type of patient, off-pump CABG demonstrated a higher survival rate at 10 years (84.8% *vs.* 75.8%, *P*=0.02) and 15 years (65.4% *vs.* 58.5%, *P*=0.01) compared to on-pump CABG. Even in patients with multivessel disease and depressed ejection fraction, five-year mortality was lower in the off-pump group (74.4% *vs.* 68.2%, *P*=0.024) and had a trend towards better survival over 10 years (63.8% *vs.* 50.4%, *P*=0.078)^[18,19]^. Much literature has reported that early mortality is similar in off-pump *vs.* on-pump CABG, and with no significant differences between the two, even off-pump, pump-assisted, and combined techniques have been compared with no differences in perioperative mortality and long-term survival^[20,21]^. In contrast, for many years it has been suggested that off-pump CABG was associated with a higher risk of early mortality than on-pump CABG^[10]^; however, some reports have described that this benefit is more evident during the first five years of follow-up and may be due to higher rates of reoperation and revascularization in patients with off-pump CABG (*P*=0.001)^[22]^. Much research on the subject has suggested that these observed differences between the two surgical approaches may be related to population factors, coronary artery anatomy, patient selection, and surgical team expertise rather than the procedure itself.

## CONCLUSION

In conclusion, this first Peruvian registry of patients treated with CABG has preliminarily demonstrated that off-pump CABG was associated with lower shortand mid-term morbidity and mortality rates, as well as better adjusted survival rates compared with on-pump CABG; however, further multicentre studies in Latin America are needed to elucidate its benefits compared with classic on-pump CABG.
